# The IL1β-HER2-CLDN18/CLDN4 axis mediates lung barrier damage in ARDS

**DOI:** 10.18632/aging.102804

**Published:** 2020-02-15

**Authors:** Xinhua Ma, Xin Yu, Qi Zhou

**Affiliations:** 1 Department of Intensive Care Unit, Xiangya Hospital, Central South University, Changsha, China; 2 Department of Pulmonary and Critical Care Medicine, Center for Respiratory Diseases, China-Japan Friendship Hospital, National Clinical Research Center for Respiratory Diseases, Beijing, China; 3 Department of Anesthesiology, First Affiliated Hospital of Hunan University of Traditional Chinese Medicine, Changsha, China

**Keywords:** acute respiratory distress syndrome, lung barrier injury, IL-1β, HER2, claudin18

## Abstract

Objective: The high mortality rate associated with acute respiratory distress syndrome (ARDS) is a major challenge for intensive care units. In the present study, we applied bioinformatics and animal models to identify core genes and potential corresponding pathways in ARDS.

Results: Using bioinformatics analysis, IL-1β was identified as the core gene of ARDS. Cell experiments showed that up-regulation of IL-1β downregulates claudin18 to promote lung barrier function damage by regulating the IL-1β-HER2/HER3 axis, further promoting the development of ARDS. This was validated in the animal models.

Conclusion: IL-1β promotes the development of ARDS by regulating the IL-1β-HER2/HER3 axis. These findings deepen the understanding of the pathological mechanisms of ARDS.

Methods: Transcription data sets related to ARDS were subjected to differential expression gene analysis, functional enrichment analysis, and receiver operating characteristic curve analysis and, so as to identify core genes in ARDS. Cell experiments were used to further explore the effects of core genes on lung barrier function damage. Animal models were applied to validate the effects of core gene in mediating biological signal pathways in ARDS.

## INTRODUCTION

Acute respiratory distress syndrome (ARDS) is a major cause of death in intensive care units (ICU) [1, 2]. It is a serious problem and challenge for clinical work in the ICU, because there have been no breakthroughs in the treatment of ARDS [3]. Therefore, there is an urgent need to identify the core genes and pathways that regulate the development of ARDS.

The pathological changes of lung tissue in ARDS are characterized by osmotic edema. Injury of alveolar epithelial cells and endothelial cell barrier function are the main pathophysiological mechanisms of osmotic pulmonary edema [4]. The increased permeability of pulmonary capillary endothelial cells and alveolar epithelial cells leads to a large amount of fluid exudation in the alveolar space, eventually leading to refractory hypoxemia that is unresponsive to conventional oxygen therapy. The connections between cells are the main components of the lung barrier. The connections between the alveolar epithelium are tight junctions, adhesive junctions, and cell desmosomes [5]. Animal experiments have shown that there is an increase in permeability of the lung epithelium, damaging to cell junctions in a lipopolysaccharide-induced (LPS-induced) ARDS model [6]. Models for lung epithelial junction injury have been established, mainly focusing on endotoxin induction, mechanical stretch, bleomycin, long-term drinking and smoking. In these models, lung epithelial junction injury was associated with expression of several signaling pathways, connections and cell membrane localizations [7]. Correlation research indicated that members of the Claudin family (Claudin1-27) were the key components of the cellular barrier tight junction complex, which regulated cell connectivity and maintains the barrier function [8]. Claudin4 and claudin18 are expressed in alveolar epithelial cells and bronchial epithelial cells, where they play important roles in the regulation of lung epithelial cell barrier function [9]. Lung epithelial cell junction damage is associated with changes in the expression and localization of the tight junction proteins claudin4, claudin18, skeletal protein ZO-1, and adhesion-linked proteins. Nevertheless, the specific regulatory mechanisms remain unclear [10].

The human epidermal growth factor receptor (HER) family is expressed in alveolar epithelial cells that regulate the epithelial cell barrier in an inflammatory environment, participating in the development of ARDS [11]. Other studies showed that IL-1β is a key inflammatory mediator of the development of ARDS [12, 13]. The IL-1β-ADAM17-NRG-1-HER2/3 signaling pathway is important for regulating the damage of the ARDS lung barrier [14]. IL-1β-mediated barrier dysfunction is dependent on activation of the HER signaling pathway. Studies have confirmed that blocking IL-1β can reduce ventilator-associated lung injury and bleomycin-induced lung injury [12, 13, 15]. However, most of the current researches only focus on the performance of macroscopic barrier damage, there are few reports on the specific causes of lung barrier damage. Tight junctions and adhesion junctions are important junctions of the epithelial cell barrier. Cellular studies have shown that the elevated lung epithelial cell permeability is due to HER2 activation, which is associated with cell junction damage caused by altered adhesion proteins [16]. Therefore, we speculated that IL-1β was involved in the damage of cell junctions and may regulate the HER2/3 signaling pathway to promote ARDS. Nevertheless, little is known about that the damage of cell junctions, especially in the damage of the ARDS lung barrier function under the inflammatory environment. The ARDS model of sepsis was constructed so as to further study the changes and regulations of tight junction proteins during lung barrier injury. This was conducive to deepening the understanding of ARDS lung barrier function damage.

In our present study, differential expression gene analysis, function enrichment analysis, and ROC curve analysis were performed on the ARDS data sets. We found that IL-1βis an important differentially expressed gene for ARDS lung barrier impairment. We constructed an LPS-induced ARDS animal model for further study. Cellular experiments further investigated the regulation of HER2/HER3-related signaling pathways and their effects on lung barrier damage.

## RESULTS

### Abnormally expressed genes in patients with ARDS and function enrichment analysis

**Figure 1:**
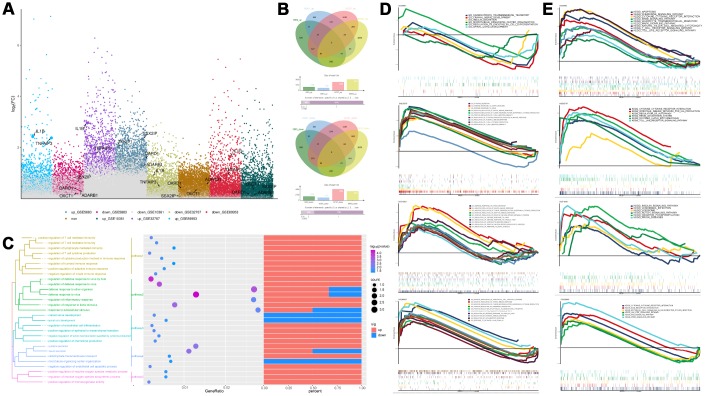
**Differentially expressed gene analysis and functional enrichment analysis.** (**A**) The differentially expressed genes in ARDS samples compared to control samples. (**B**) Cluster analysis showed the expression patterns of the common up-regulated or down-regulated genes in the four data sets can distinguish ARDS and control. (**C**) Functional enrichment analysis of biological processes for the common the common up-regulated and common down-regulated genes in the four data sets. (**D**, **E**) Gene Set Enrichment Analysis results.

Compared with the control group, there were 2883, 2051, 7713, and 8152 differentially expressed genes (DEGs) in the GSE5883, GSE10361, GSE32707, and GSE89953 d, respectively (Figure 1A, 1B). The expression patterns of the common DEGs could distinguish ARDS from control (Supplementary Figure 4). These common DEGs were further subjected to enrichment analysis (Figure 1C). In biological process (BP), these common DEGs may involved in various inflammation-related BPs, such as cell-mediated immunity, regulation of inflammatory responses, regulation of host defense against viral defense, viral defense response and endocrine cytokine secretion. In KEGG pathways, the synthesis and degradation of ketone compounds and cytokine-cytokine receptor interactions are significantly enriched in ARDS. These may be the key BPs and pathways in the development of ARDS. The results of GSEA enrichment analysis validated the above BPs (Figure 1D) and pathways (Figure 1E).

### The core gene IL-1β is generally up-regulated in ARDS patients

**Figure 2:**
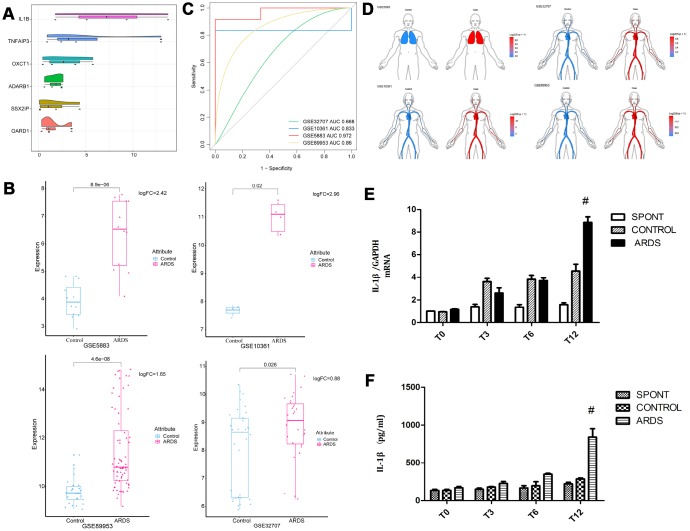
**Expression of core gene IL-1β.** (**A**) The differentially expressed genes were scored according to the degree of similarity between genes and genes. (**B**) IL-1β appeared in the four data sets of ARDS Significantly high expression. (**C**) ROC curve analysis indicated IL-1β can distinguish ARDS and control in the four data sets. (**D**) IL-1β was up-regulated in lung and whole blood. (**E**) The concentration of IL-1β in lung tissue in patients with ARDS was significantly elevated. (**F**) The concentration of IL-1β in lung tissue and bronchoalveolar lavage fluid in patients with ARDS was significantly elevated. ^#^, P < 0.05.

Six genes (IL-1β, TNFAIP3, ADARB1, OXCT1, SSX2I, and OARD1) with the most average functional similarity were considered as the hub genes (Figure 2A). IL-1β was up-regulated (Figure 2B) in all the four datasets. ROC curve analysis indicated that IL-1β was a biomarker of ARDS (Figure 2C), up-regulated both in lung and blood circulation (Figure 2D). Studies had confirmed that IL-1β was a key inflammatory mediator in the development of intrapulmonary inflammatory response, which promoted the development of pulmonary epithelial barrier dysfunction in chronic inflammation [12, 13]. Our preliminary experiments had confirmed that IL-1β was significantly elevated in the lung tissue and bronchoalveolar lavage fluid in patients with ARDS (Figure 2E, 2F), suggesting that IL-1β may have a crucial role in the development of ARDS lung barrier injury.

### IL-1β alters the permeability of lung epithelial cells BEAS-2B by HER2 and affects cell-to-cell junctions

**Figure 3:**
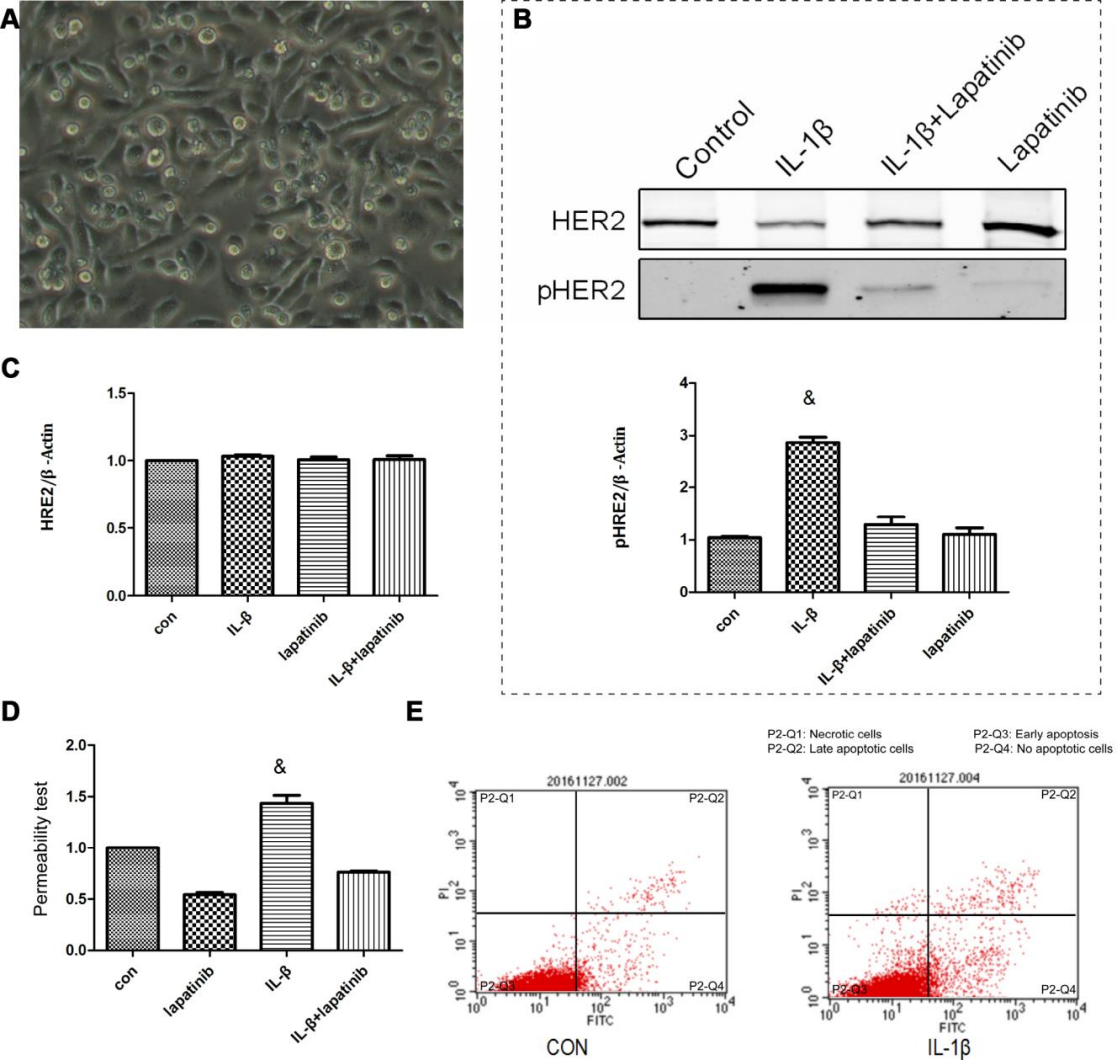
**The lung epithelial cells were divided into 4 groups.** Control group, IL-β group, lapatinib group and IL-β+lapatinib group. (**A**) Human lung epithelial cells were cultured in vitro, and the cells were adherently grown in culture flasks. Generally, the growth was good, the nucleus was located in the center of the cells, and the cytoplasm was extended outward. Once polygons grow irregularly. (**B**) The pHER2 were detected in lung epithelial cells using western blot method. Compared with the control group, the expression of pHER2 was significantly up-regulated in IL-1β group. ^&^, P < 0.01. (**C**) The expression of HER2 was not significantly different in the four groups. (**D**) Compared with the control group, the permeability of epithelial cells increased after 6 hours of IL-1β treatment. There was no significant difference between the other groups. ^&^, P < 0.01. (**E**) Under the action of IL-1β, there was no increase in apoptosis.

Human epidermal growth factor receptor (HER) is expressed in alveolar epithelial cells, involved in the process of lung epithelial cell injury and repair. IL-1β activates the HER2 signal pathway via HER2 phosphorylation, promoting the further development of ARDS [14]. Nevertheless, it was still unclear about the regulation mechanism of HER2 by IL-1β. Therefore, we cultured human lung epithelial cells BEAS-2B cells (Figure 3A) in vitro. Expression levels of HER2 and PR2 were measured using western blot. Compared to the control group, the expression of pHER2 was significantly greater in the IL-1β group (P < 0.05) (Figure 3B), and was not significantly different in the other three groups (P > 0.05) (Figure 3C). This suggests that IL-1β is involved in the HER2/HER3 signal transduction pathway. Up-regulated expression levels of pHER2 activate the downstream pathway, affecting the development of ARDS. IL-1β expression leads to increased lung epithelial permeability (Figure 3D), with reduced permeability after the addition of the HER2 blocker lapatinib. This finding suggests that IL-1β affected the development of ARDS by directly regulating the HER2/HER3 signal transduction pathway. We observed that apoptosis levels in each group were different (P <0.05) (Figure 3E). This suggests that, under the action of IL-1β, there was not only no increase in apoptosis in cells, but also no difference in the apoptosis of lung epithelial cells. This further suggests that damage to the lung barrier function had nothing to do with apoptosis of lung epithelial cells, but may be linked to damage between cells and cells.

### IL-1β regulates the expression and localization of claudin4/18 by HER2

In lung epithelial cell junctions, IL-1β expression leads to lung epithelial cell junction damage by changes in the expression and localization of the tight junction proteins clauin4, clauin18, skeletal protein ZO-1, and adhesion-linked proteins [10]. Claudin4 and claudin18 are mainly expressed in alveolar epithelial cells and bronchial epithelial cells, playing an important role in the regulation of lung epithelial cell barrier function [9]. Changes in their expression levels and localization might affect lung barrier function damage.

**Figure 4:**
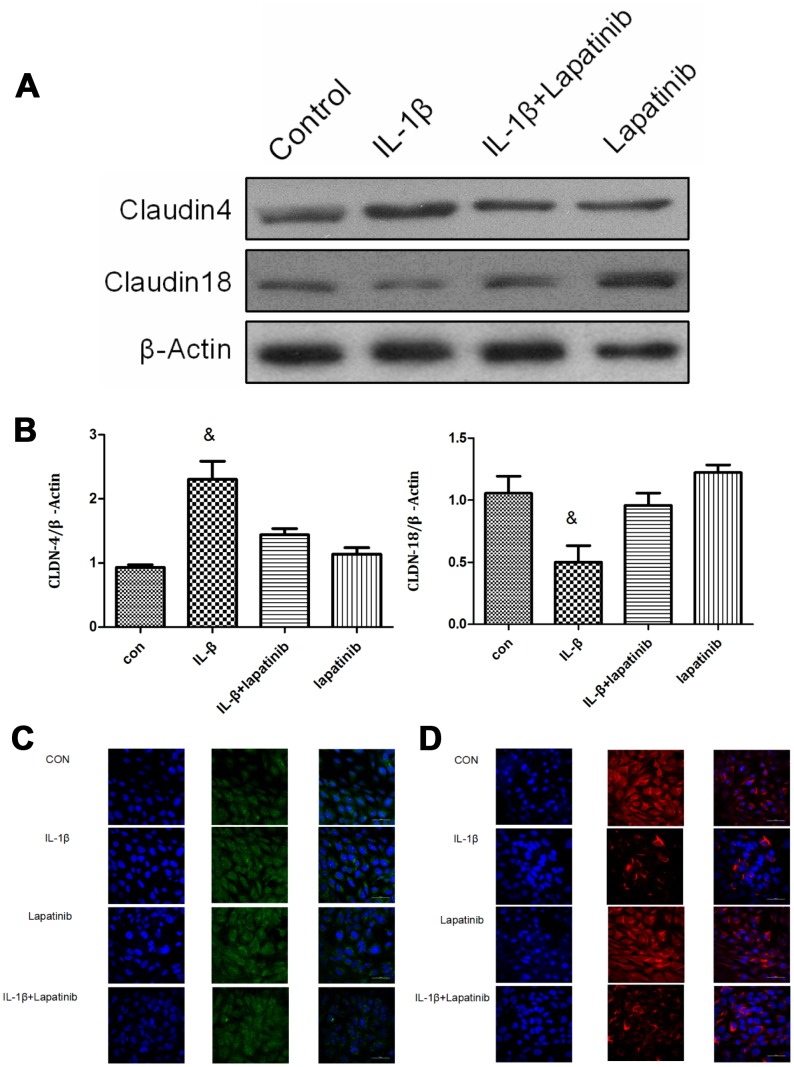
**Analysis of claudin4/18 expression and localization.** (**A**, **B**) The expression of claudin4 expression was significantly up-regulated while the expression of claudin18 was significantly down-regulated in the IL-1β group. ^&^, P < 0.01. (**C**) Claudin4 immunofluorescence was increased significantly in the cell membrane localization. (**D**) Claudin18 immunofluorescence results was reduced significantly in cell membrane localization.

The expression of claudin4 was upregulated in the IL-1β group (P < 0.05), however, there was no significant difference between lapatinib group and IL-1β + lapatinib group (P > 0.05) (Figure 4A, 4B). Compared with the control group, the expression of claudin18 in the IL-1β group was significantly lower (P < 0.05), while there was no significant difference between lapatinib group and IL-1β+Lapatinib expression (P >0.05). After the addition of the HER2 blocker lapatinib, there was no difference in the expression of claudin4 and claudin18, suggesting that the HER2 blocker lapatinib blocked the effect of IL-1β on the expression of claudin4 and claudin18. Combined with the results of immunofluorescence (Figure 4C), claudin18 protein fluorescence was significantly lower in terms of cell membrane localization, with decrease of fluorescence intensity. Claudin4 expression was the opposite (Figure 4D). These findings further verify that IL-1β activates HER2/HER3-related signaling pathways. It regulates the expression and spatial localization of claudin4 and claudin18, resulting in increased barrier permeability of lung epithelial cells.

### Verification of claudin4/18 expression and localization changes in ARDS model

**Figure 5:**
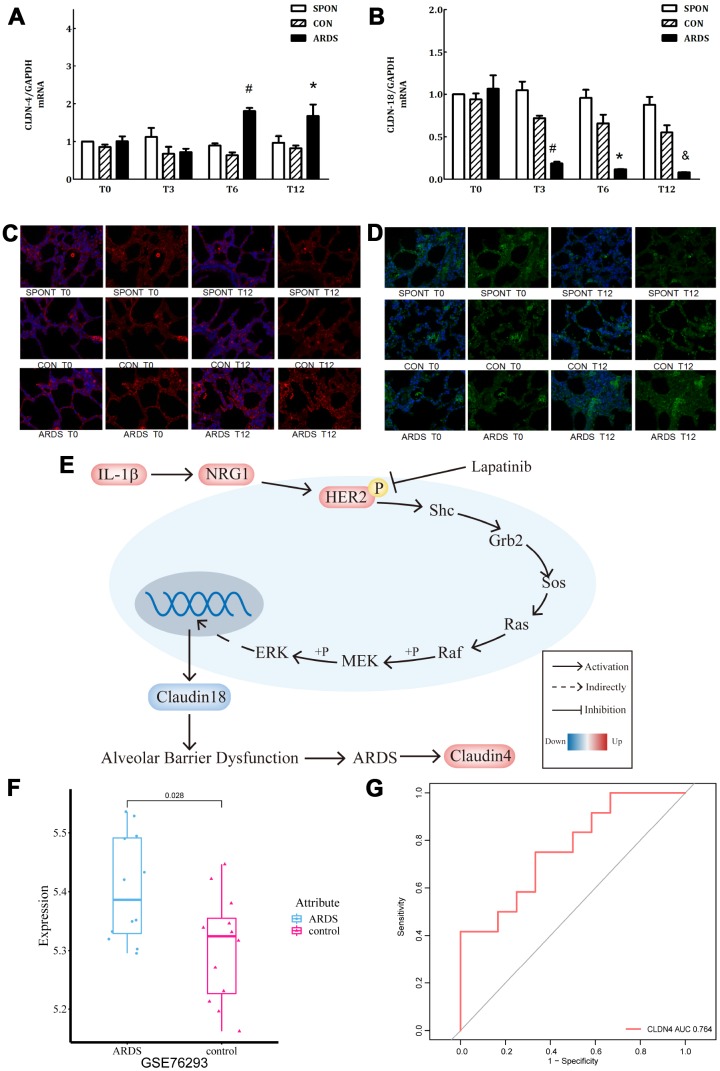
**Further verification of claudin4/18 expression and localization in the ARDS model.** (**A**) Comparison of expression levels of claudin4, claudin4 expression significantly increased at 8 hours and 12 hours in ARDS. (**B**) Claudin18 expression in lung tissue decreased significantly at 3 hours, 6 hours, and 12 hours of ARDS. (**C**). Three groups of 0, 3, 6 and 12 hours of lung tissue. Claudin4 immunofluorescence, purple fluorescence is the nucleus, red fluorescence is claudin4 protein, and the fluorescence and fluorescence intensity around the cells are compared. The fluorescence around the cells in the ARDS group was significantly stronger than that in the other groups at 12 hours. The expression of claudin4 protein was increased during the induction of lung tissue in the ARDS model. (**D**) The lung tissues of three groups at 0, 3, 6 and 12 hours showed immunofluorescence. The nucleus was stained with blue fluorescence and the protein was stained with red fluorescence. The 12-hour peripheral fluorescence of ARDS group was significantly weaker than that of other groups. During the induction of ARDS model, the expression of lung tissue decreased and the location of protein on cell membrane decreased; (**E**) IL-1β activated HER2 signaling pathway by up-regulating NRG1, which regulates the expression of claudin18 protein, promoting the development of ARDS. Furthermore, in turn, this would initiate a compensatory protective mechanism, promoting the expression of claudin4, strengthening the repair of lung barrier function. The series of reactions would be reversed by the addition of lapatinib, a HER2 blocker. (**F**) Claudin4 was up-regulated in ARDS compared to control in GSE76293. (**G**) ROC curve results indicated that claudin4 can distinguish ARDS and control in GSE76293.

In the ARDS model, claudin4 expression in lung tissue increased significantly at 6 and 12 hours of ARDS (P < 0.05), while claudin18 expression decreased significantly at 3, 6, and 12 hours (P < 0.05), however, there was no significant difference in other groups (P > 0.05) (Figure 5A, 5B). This finding suggests that up-regulated claudin4 and down-regulated claudin18 expression promotes the development of ARDS [5]. Several studies have demonstrated a sepsis-induced ARDS model characterized by decreased lung barrier function and pulmonary edema. Previous studies have shown that increased claudin4 levels are associated with increased lung water clearance and reduced damage to the physiological lung barrier [17–19]. The results of immunofluorescence showed that, compared with other groups, the immunofluorescence of claudin4 protein increased at 12 hours, while that of claudin18 protein was the opposite (Figure 5C, 5D). This finding suggests that down-regulation of claudin18 may result in the destruction of the barrier function of the lung epithelial cells, leading to the formation of pulmonary edema. The increase in claudin4 expression levels in the ARDS model may be related to a compensatory function of the body. We demonstrated the role of IL-1β in the HER2 signaling pathway (Figure 5E). This is an important regulator of lung barrier function, further activating the HER2 signaling pathway by NRG1. The autophosphorylation of HER2 ultimately reduced the expression of claudin18 protein, with the result of the damage in lung epithelial barrier, further promoting the development of ARDS. The incidence of ARDS triggers compensatory protective mechanisms, promoting the expression of claudin4, further strengthening the functional repair of lung barrier. The series of reactions is reversed by the addition of the HER2 blocker lapatinib, further suggesting that the HER2 signaling pathway is a critical pathway. Claudin4 was down-regulated (Figure 5F) in GSE76293. ROC curve analysis suggested that claudin4 is a biomarker of ARDS (Figure 5G).

## DISCUSSION

ARDS is one of the most serious diseases with high mortality in clinical practice. The primary pathophysiological mechanism is lung barrier damage. The lung barrier includes the lung epithelial cell barrier and the endothelial cell barrier. The epithelial cell barrier is composed of the connection between the epithelial cells and the cells, which plays an important role in regulating the function of the lung epithelial cell barrier, water and electrolyte transport, as well as information transmission. Cell junctions include tight junctions, adhesive junctions and bridging junctions. The three cell junctions are interdependent and coordinated to maintain the stability of the lung epithelial barrier function.

We found that IL-1β expression was generally significantly higher in ARDS patients by analyzing the related gene expression profiles, consistent with the results of a previous study. We confirmed that highly expressed IL-1β plays a significant role in ARDS. Previous studies had confirmed that the HER receptor family regulates lung epithelial barriers repairing in lung epithelial damage, especially in inflammatory conditions. It has mainly four sub-types, namely HER1, HER2, HER3 and HER4, which exert different physiological effects by binding to their respective ligands. HER2 is not a self-ligand that needs to activate its downstream signaling pathway by its own phosphorylation [14, 20]. In the process of culturing lung epithelial cells, adding IL-1β up-regulates the expression of pHER2 and increases the permeability of lung epithelial cells; however, it does not cause the increase of apoptosis. By adding the HER2 blocker lapatinib, we observed that both pHER2 expression and lung epithelial cell permeability decreased. This may confirm that IL-1β upregulated pHER2 expression and lung epithelial permeability by regulating the HER2/HER3 signal transduction pathway. It has been confirmed in previous studies that the HER2 signal transduction pathway is crucial for the lung barrier and adhesion structure damage. Under normal circumstances, apoptosis is programmed cell death that eliminates senescent cells, avoiding the pathological state that may occur due to cell necrosis. Under various external stimuli, intracellular control switches determine the apoptotic state of cells [21]. Sepsis produces a large number of inflammatory mediators and pathophysiological responses. The activation and apoptosis of inflammatory cells participated in every stage of ARDS. Inflammatory mediators may cause apoptosis in lung epithelial cells, further leading to pulmonary epithelial barrier dysfunction [22]. In our study, despite the fact that IL-1β is a key inflammatory factor, the apoptosis of lung epithelial cells did not increase after we added IL-1β. This suggests that the damage of lung epithelial cells may be related to the damage of cell-cell connections. In lung epithelial cell junctions, it can lead to lung epithelial cell junction damage by changing the expression and localization of the tight junction proteins claudin4, claudin18, skeletal protein ZO-1, and adhesion-linked proteins [10]. Tight junction proteins are a key to the tight junctional complex, which are important structures for maintaining the function of the lung epithelial barrier. Recent studies have shown that claudin family members (claudins 1–27) are key components of the cellular barrier tight junction complex, regulating cell junctions and maintain the barrier function [7, 8, 23]. Claudin4 and claudin18 are expressed in alveolar epithelial cells and bronchial epithelial cells, playing important roles in the regulation of lung epithelial cell barrier function. In vitro cytology experiments showed that down-regulation of claudin18 protein led to destruction of tight junctions in alveolar epithelial cells [24]. Another study found that, in rats with knocked out claudin18 gene, there was lower lung barrier function and increased permeability. Further study found that the expression of claudin4/3 protein increased in claudin18 knockout rats [17]. All these results confirm that claudin18 protein is not only the key molecule for tight connection of lung barrier epithelial cells, but also regulates the function of related proteins on lung epithelial cells. In this study, we established the animal model of ARDS for further study. We confirmed that the expression of claudin4 protein increased, while that of claudin18 was the opposite. We conclude that the downregulation of claudin18 may lead to the destruction of the barrier function of the lung epithelial cells, generating pulmonary edema. According to previous studies, increased claudin4 levels were associated with better lung water clearance and less damage of the physiological lung barrier [17–19, 25]. The increase in claudin4 expression in the ARDS model may be due to an increase in compensatory claudin4 expression in the body following impaired lung barrier function in ARDS. In normal lung epithelial cells, the expression of claudin4 was significantly up-regulated while that of claudin18 was down-regulated in the IL-1β group. There was no difference in the expression of claudin4 and claudin18 in which lapatinib was added. This suggests that the HER2 blocker lapatinib blocks IL-1β the effects of claudin4. The effect of claudin18 expression further demonstrated that IL-1β activates the HER2/HER3-associated signaling pathway to regulate the expression and spatial localization of claudin4 and claudin18, resulting in increased barrier permeability in lung epithelial cells.

Although the results of this study have deepened the understanding of the development of ARDS, there remain some limitations. First, the sample size of this study was small, although animal experiments and cell experiments have verified the effect of IL-1β on the HER2 signal transduction pathway. Whether it can be used as a biomarker for ARDS remains to be validated in a larger data set.

In conclusion, IL-1β may promote the development of ARDS through regulation of the IL-1β-HER2/HER3 axis. The results of the resent study have deepened the understanding of the pathological mechanism of ARDS.

## MATERIALS AND METHODS

### Bioinformatics analysis

### Data preparation

The datasets were downloaded from Gene expression omnibus (GEO) [26], including GSE5883, GSE10361, GSE32707, and GSE89953. The human lung microvascular endothelial cell gene expression profiles of GSE5883 contain 12 ARDS and 12 controls based on GPL570. The platelet gene expression profiles of GSE10361 contain six acute lung injury and three controls based on GPL96. The whole blood expression profiles of GSE32707 contain 31 ARDS and 34 controls based on GPL10558. The peripheral blood monocytes gene expression profiles of GSE89953 contain 68 ARDS and 26 controls based on GPL6883. Thus, 194 samples were brought into in total, including 117 ARDS and 75 control samples. The gene expression profiles were normalized respectively using the *normalizeBetweenArrays* function in the limma package [27] in R. If a gene corresponded to multiple probes, the average value of these probes was considered as the expression value of the corresponding gene.

### Screening differentially expressed genes (DEGs) and core genes

The limma package was applied to differential expression analysis in the four data sets. P adjusted by false discovery rate(FDR) <0.05 and |logFC | >1 were set as the threshold. Compared to control group, if a gene was up-regulated or down-regulated in all the four data sets, it was defined as a common DEG. We ranked the common genes inside the interactome by the average functional similarities between the gene and its interaction partners. Genes with a higher average functional similarity were considered as the more crucial genes [28].

### Receiver operating characteristic (ROC) curve analysis

The pROC package [29] was applied for ROC curve analysis was applied for ROC curve analysis for common DEGs to assess their diagnostic ability for ARDS.

### Enrichment analysis

Gene Ontology (GO) biological processes (BPs) and Kyoto Encyclopedia of Genes and Genomes (KEGG) pathway enrichment analysis were performed for the common DEGs. GO and KEGG enrichment analysis were performed to be used by clusterProfiler package [30] in R. P adjusted by FDR <0.05 was considered significant.

### Gene set enrichment analysis (GSEA)

GSEA [31] was applied to explore the related BP and KEGG pathway and performed using GSEA JAVA software (https://software.broadinstitute.org/cancer/software/gsea/wiki/index.php/Main_Page). The c2.cp.kegg.v6.2.symbols.gmt [32] gene sets were to be as the reference gene set. Nom P <0.05 was considered significant.

### Establishment and identification of animal models

Sources of experimental animals: all animals were from the Department of Zoology of Central South University. New Zealand rabbits (2.5-3.5 kg) were raised in the Animal Experimental Center of Xiangya Third Hospital. The animals were given sterile water and food in a standard environment. The study has been approved by the Ethics Committee of the Animal Protection Association of the Third Xiangya Hospital (No.201504492). Supplementary Table 1 was provided in detail.

### Experimental method

After fixation, the experimental animals were punctured and anesthetized with pentobarbital sodium. Tracheotomy was performed under local anesthesia. No. 3 tracheal tube was inserted, connected to assist breathing. Left femoral vein catheterization was performed to monitor blood pressure, pulse and body temperature (Supplementary Figure 1).

The experimental animals were kept warm using external heating equipment when the operation was completed. After vital signs are stabilized for 30 minutes, 300 μl of femoral artery blood was taken to check the arterial blood gas analysis. If there was no obvious abnormality in blood gas parameters, the time point was defined as T0, and then every 3 hours (marked as T3, T6, T9, and T12 time points) and recorded as T3, T6, T9, and T12 data, respectively.

### Constructing an LPS-induced ARDS model

We used the method of Matute-Bell et al. [6]: intratracheal injection of 1 mg/ml LPS 2 mg/kg. Rabbits are very sensitive to hypoxia. When constructing the model, we used ventilators to change the connector, without interruption of mechanical ventilation. Modeling qualified indicators were as follows: 1, the lung tissue pathological damage score; 2, alveolar capillary permeability damage: increased lung water, increased alveolar protein concentration, lung tissue wet and dry weight; 3, intrapulmonary inflammatory response; 4, physiological evidence of insufficiency; the above three or more occurrences within 24 hours prove that the ARDS model was successfully established. We monitored hemodynamic parameters (Supplementary Figure 2).

### Histopathology of model lung tissue

According to the experimental procedure, the animals were sacrificed by bloodletting at the end of the experiment. We then removed the lung lobes from the chest cavity. The left lung was subjected to bronchoalveolar lavage, and the right lower lobe was examined for histopathology and molecular biology. We measured wetness and weight ratio using the right middle lobe (Supplementary Figure 3).

### Lung tissue immunofluorescence assay

Lung tissue was embedded in paraffin and sectioned. Then immunofluorescence tests were carried out. First, after paraffin sections were dewaxed, the antigen was repaired, followed by spontaneous circular fluorescence quenching. Then the serum was sealed, the first antibody and the second antibody were added successively. After using DAPI to dye the nucleus and seal it, we obtained photographs (ultraviolet excitation wavelength 330–380 nm, emission wavelength 420 nm; FITC green excitation wavelength 465–495 nm, emission wavelength 515–555 nm; Cy3 red excitation wavelength 510-560, emission wavelength 590 nm).

### Claudin4/18 and IL-1β mRNA expression in lung tissue

RNA specimens were measured for purity using an extraction kit (EZNA Total RNA Kit II Omega Bio-tek USA). ELISA was used to measure Claudin4/18 and IL-1β concentrations in lavage fluid at the end of the test.

### Effect of IL-1β on lung epithelial cells

Human lung epithelial cells (BEAS-2B) were cultured in vitro, divided into four groups: control group, IL-β group, lapatinib group and IL-β+lapatinib group. IL-1β was a pro-inflammatory mediator and lapatinib is a blocker of the HER2 signaling pathway. According to the ATCC guidelines, BEAS-2B cells were routinely cultured in BEGM medium and cultured in an incubator with an environment of 5% CO2, 37°C, with saturated humidity. Every 6 days, the subculture was carried out once, and the fluid was changed 3-5 days after subculture. Apoptosis was detected in cells, furthermore, the cell permeability was detected by fluorescein labeling. The expression and localization of claudin4/18 on cell membrane were detected by immunofluorescence, and then the expression of IL-1 β and claudin4/18 were measured using western blot.

### Statistical analysis

Statistical analysis was performed using R software (version 3.5.3) and GraphPad Prism 6.0. The Kolmogorov–Smirnov method was applied to test data normality. The homogeneity of the variance was tested using Levene's method. Results were expressed as mean and standard deviation of normally distributed data. Mean ± standard deviation was used for verification. The median (full range) was applied to describe the centralized and discrete trend of non-conforming normal distribution data. The Mann–Whitney U nonparametric test was used for comparing inter group. The Chi square test was used for the comparison of count data between groups. Repeated measurement ANOVA was used to compare data of various time points among groups. P < 0.05 defined statistically significant differences.

### Ethics approval

The Ethics Committee of the Animal Protection Association of the Third Xiangya Hospital (No.201504492) (Changsha, China) approved all animal experimental procedures.

## Supplementary Material

Supplementary Figures

Supplementary Table 1
