# Supplementary material for: The IL1β-HER2-CLDN18/CLDN4 axis mediates lung barrier damage in ARDS
Source: Aging (Albany NY). 2020 Feb 15;12(4):3249–65. doi: 10.18632/aging.102804 (PMC7066891; doi:10.18632/aging.102804)
Supplement: Supplementary Figures [file aging-12-102804-s002..pdf]

SUPPLEMENTARY FIGURES

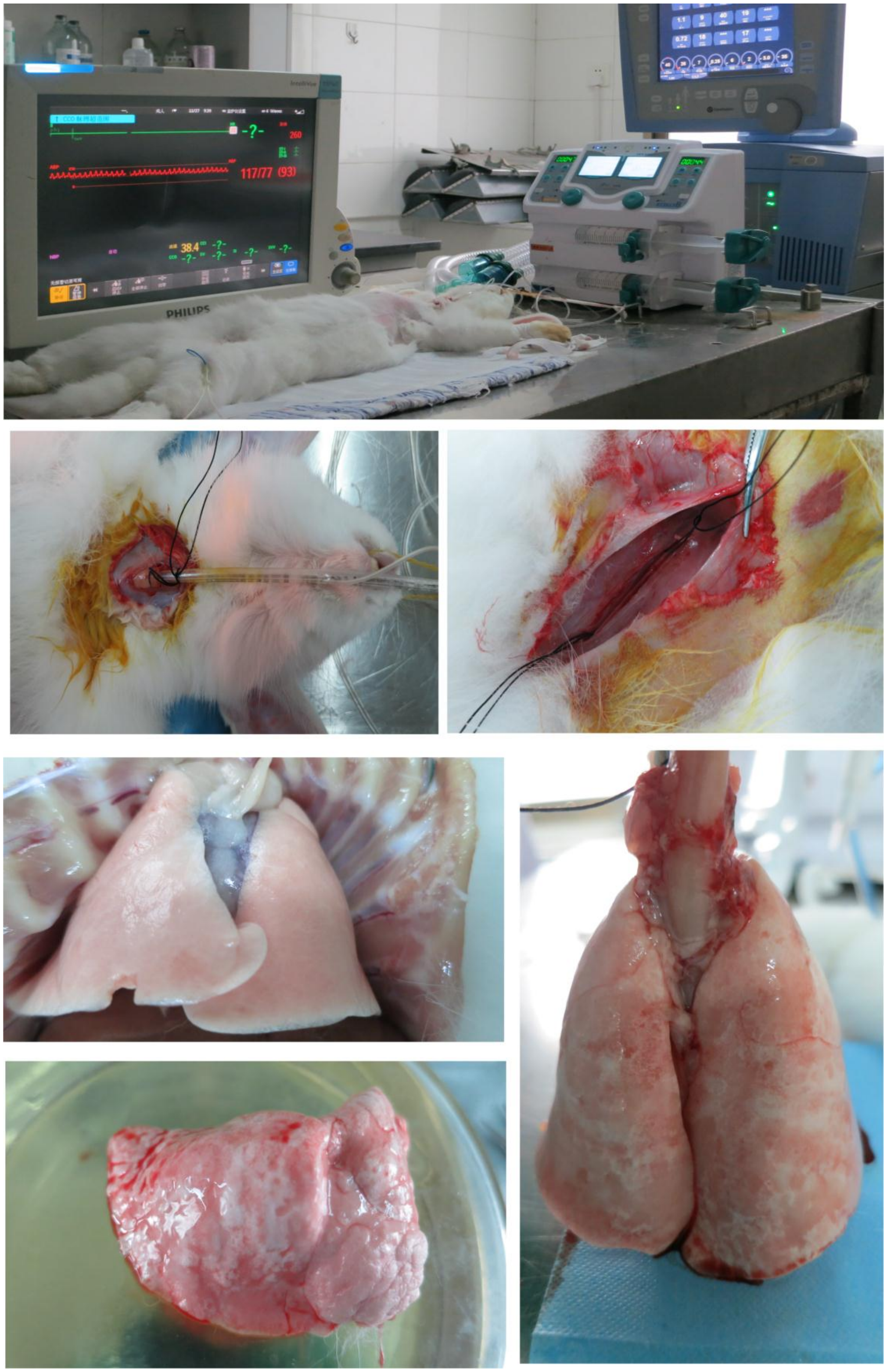

Supplementary Figure 1. Experimental procedure. Puncture of the ear vein, local anesthesia for tracheotomy, catheterization, ventilator assisted breathing, open left femoral vein catheterization, and monitoring blood pressure, pulse and body temperature.

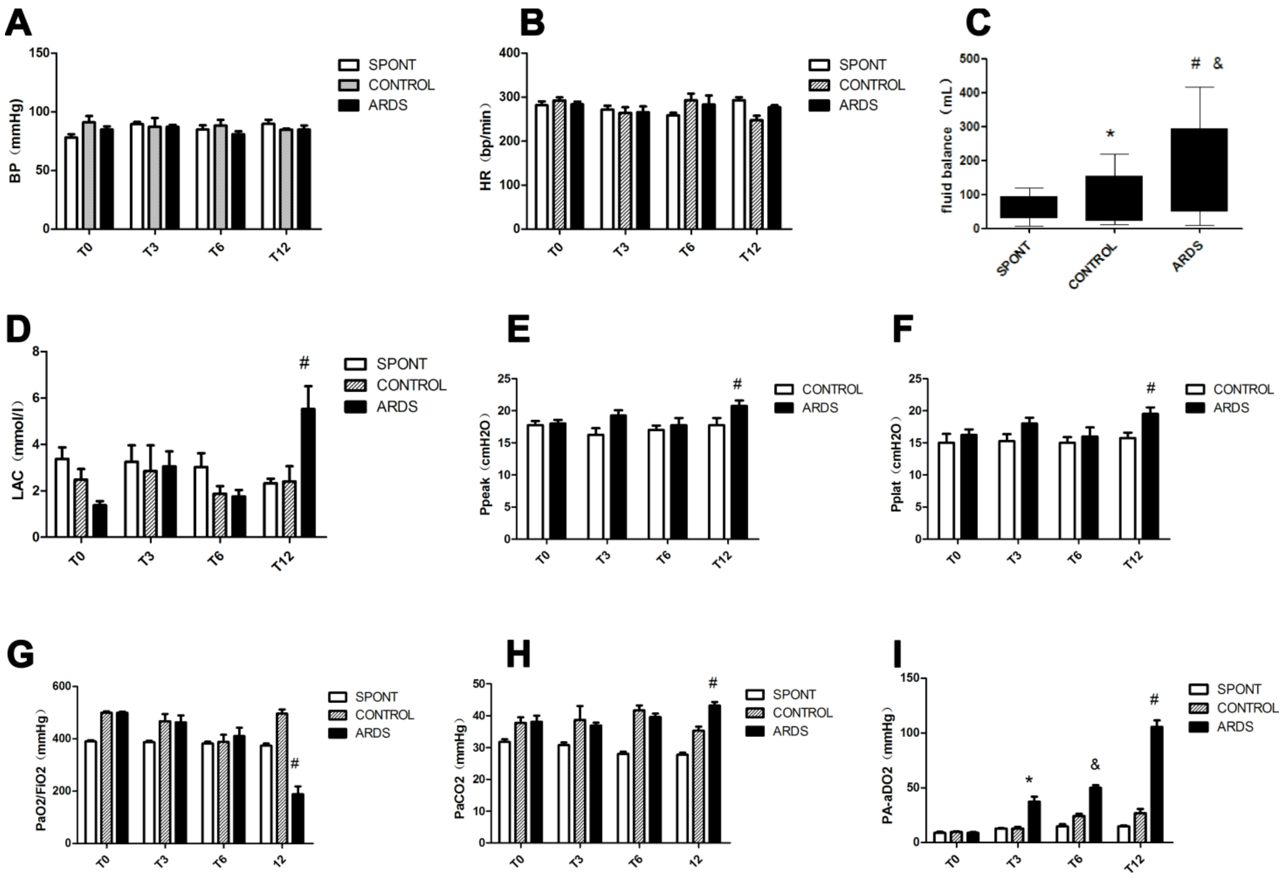

Supplementary Figure 2. After the animal's vital signs are stable, blood gas analysis and blood tests are performed. If there is no obvious abnormality in blood gas, the time point was defined as T0, and then every 3 hours (marked as T3, T6, T9, T12 time points) is recorded as T3. T6, T9, T12 data. (**A**) Three groups of 0, 3, 6 and 12 hour blood pressure, heart rate comparison, (**B**) three groups of liquid balance, three groups of 0, 3, 6 and hour lactic acid comparison, (**C**) three groups of 0, 3, 6 and 12 hour peak pressure Comparison of platform pressure, (**D**) Comparison of three groups of 0, 3, 6 and 12 hour blood gas analysis.

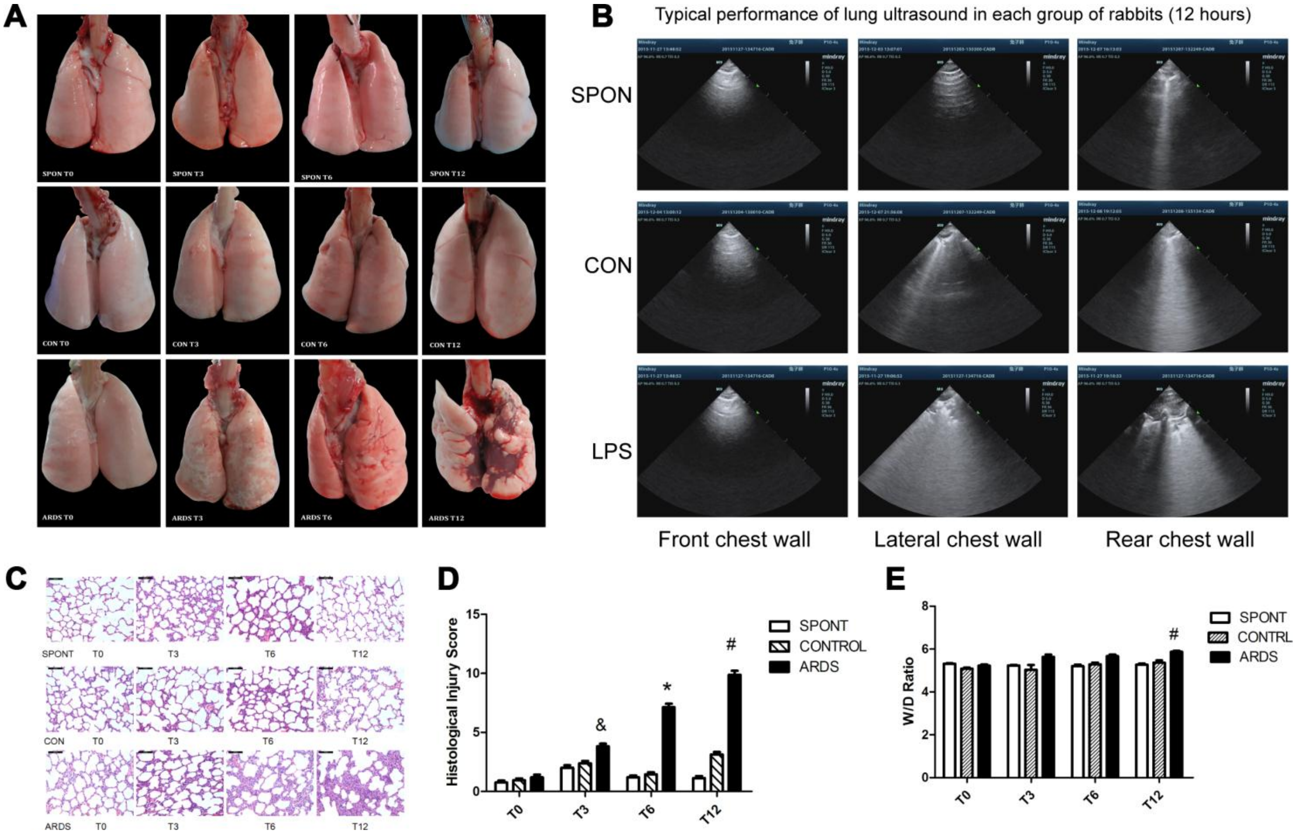

Supplementary Figure 3. Observation of animal model specimens. (**A**) Three groups of 0, 3, 6 and 12 hour of gross lung tissue. (**B**). Typical pulmonary ultrasound (12 hours). (**C**). Three groups of lung tissue 0, 3, 6 And 12-hour HE staining (×200). (**D**) Three groups of 0, 3, 6 and 12 hour light microscopic pathological damage scores of lung tissue (**E**). Comparison of lung tissue wet and dry weight at different time points in three groups of animals.

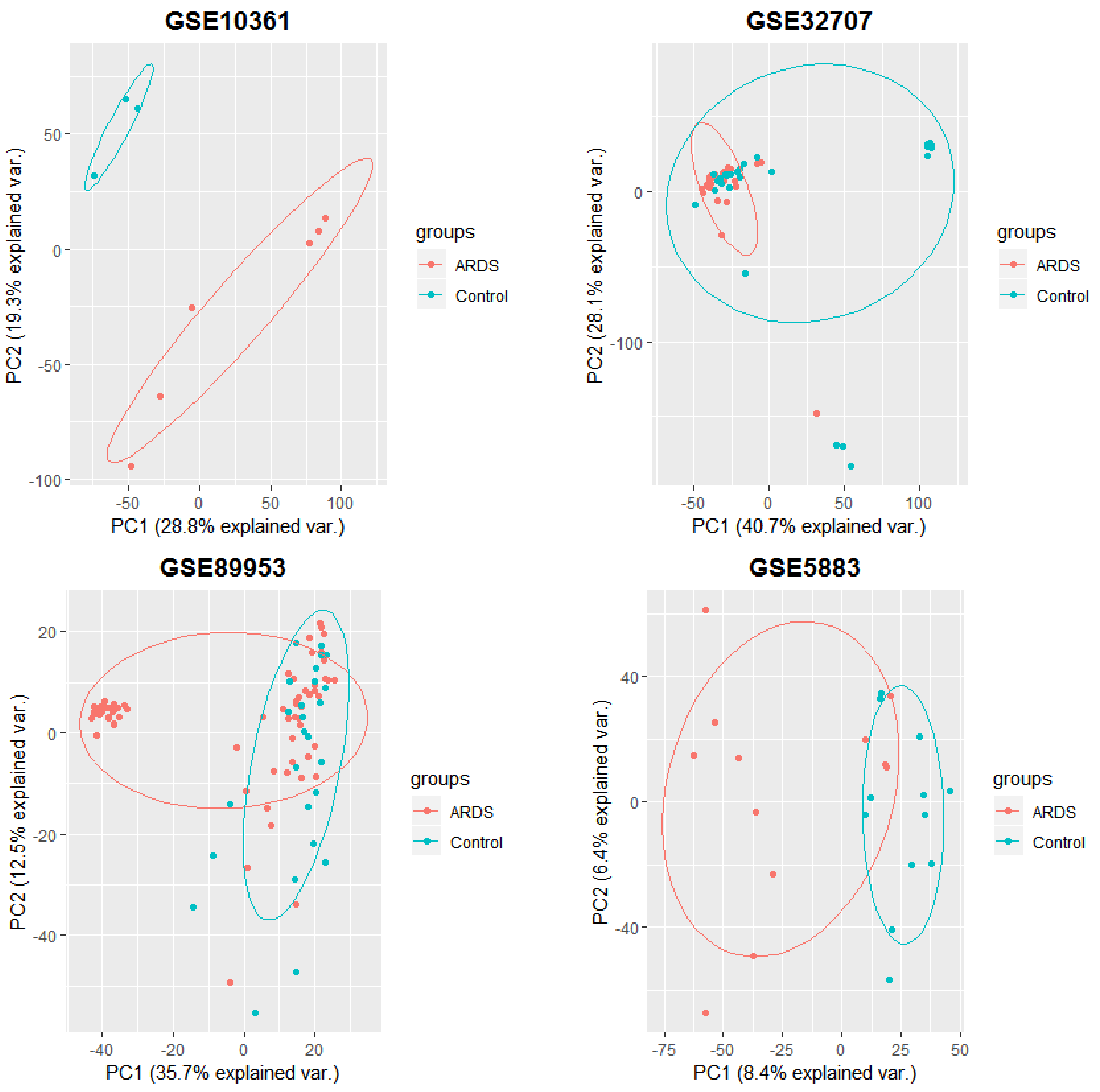

Supplementary Figure 4. Principal component analysis of ARDS in gene expression profiling.
