# Supplementary material for: The IL1β-HER2-CLDN18/CLDN4 axis mediates lung barrier damage in ARDS
Source: Aging (Albany NY). 2020 Feb 15;12(4):3249–65. doi: 10.18632/aging.102804 (PMC7066891; doi:10.18632/aging.102804)
Supplement: Supplementary Table 1 [file aging-12-102804-s001..pdf]

SUPPLEMENTARY TABLE

Supplementary Table 1. Comparison of general conditions between spontaneous breathing group, control group and ARDS group.

Group	SPON	CON	ARDS
Number	16	16	16
Weight (kg)	3.05±0.20	2.96±0.37	3.12±0.19
Tidal volume (ml/kg)	-	10	10
PEEP (cmH_2O)	-	2	2
Oxygen concentration	21%	30%	30%
Vasoactive drug (n)	0	2	5
